# Multidisciplinary Approach for the Treatment of Horizontal Root-Fractured Maxillary Anterior Teeth

**DOI:** 10.1155/2014/472759

**Published:** 2014-11-17

**Authors:** Berkan Celikten, Ceren Feriha Uzuntas, Reza Safaralizadeh, Gulbike Demirel, Semra Sevimay

**Affiliations:** ^1^Department of Endodontics, Faculty of Dentistry, University of Ankara, Besevler, 06500 Ankara, Turkey; ^2^Department of Restorative Dentistry, Faculty of Dentistry, University of Ankara, Besevler, 06500 Ankara, Turkey

## Abstract

Dental trauma can lead to a wide range of injuries of which crown and root fractures are examples. Crown-root fractures often need complex treatment planning. This case report describes the use of MTA in the multidisciplinary management of a patient with a horizontally fractured central incisor and luxation in a different central incisor. A 42-year-old female patient presented within 1 h of receiving direct trauma to her maxillary area. Clinical examination revealed that the right and left maxillary central incisors presented mobility and sensitivity to percussion and palpation but no sensitivity to thermal stimulations. Occlusal displacement with extrusion in the left maxillary central incisor and luxation in the right maxillary central incisor was observed. Radiographic examination revealed horizontal root fracture at the apical third of the left maxillary central incisor. Root fracture in the right maxillary incisor was not observed. Endodontic and aesthetic restorative treatments were completed. MTA showed a good long-term outcome when used in root-fractured and luxated teeth. In addition, composite resin restoration provided satisfactory aesthetic results even after 15 months.

## 1. Introduction

Traumatic dental injuries are among the main causes of emergency treatment in dentistry and occur frequently as a result of adverse incidents such as falls, traffic accidents, impact sports, and fights [1]. Horizontal root fractures due to high force exerted on the root are characterised by fractures of the hard structures of the root in the coronal, mid, or apical portions. Fractures may involve the pulp, dentin, and cement and are associated with injuries of the periodontal ligament, supporting alveolar bone, and tooth separation. The apical segment of the tooth is rarely displaced; however, the coronal segment is often displaced [2, 3].

Horizontal fractures usually occur in the central maxillary incisors. These fractures subsequently lead to aesthetic, functional, and phonetic problems [4], and healing from fractures can be complicated. Andreason et al. [5] reported four different types of healing in root fractures: (a) healing with calcified tissue, (b) healing with conjunctive tissue interposition, (c) healing with bone and conjunctive interposition between the fragments, and (d) healing with granulation tissue between the fragments.

Pathological complications encountered in horizontally fractured teeth might include pulp necrosis, root canal obliteration, external and internal root resorption, inflammation around the fracture, and periapical inflammation [6, 7].

Diagnosis of the horizontal root fracture is based on clinical and radiographic examinations. Clinical examinations reveal whether the crown is normal or extruded, depending on the localisation of the fracture (apical/mid/cervical third), tooth mobility, and presence/absence of pain to palpation of the soft tissues and percussion of the teeth [2, 8]. Radiographic examination must be performed carefully to observe the fracture line. A conventional periapical radiograph and two additional periapical radiographs (one with a positive angulation of 15° to the fracture line and one with a negative 15° angulation) should be taken. In addition, occlusal radiographs may be required to disclose fractures in the apical third of the root [9–11].

Horizontal root fractures can be treated by repositioning the coronal fragment and affixing a semirigid or rigid splint to the adjacent sound teeth. The splint should be maintained depending on the localization of the fracture and the vitality of the traumatised tooth should be checked for the following 2-3 months. In most cases in which the pulp in the apical fragment remains vital, endodontic treatment of the coronal fragment is sufficient. However, if the apical pulp becomes necrotic during this period, endodontic treatment should be carried out using one of the following treatment options: endodontic treatment of both fragments, endodontic treatment of the coronal fragment and surgical removal of the apical fragment, extraction of the coronal fragment and endodontic treatment, and orthodontic extrusion of the apical fragment [12].

Mineral trioxide aggregate (MTA) has several potential clinical applications owing to its superior sealing property and biocompatibility [11]. MTA is highly effective in pulp capping after pulpotomy and sealing the pathways between the root canal system and the external surface of the tooth. Several case reports have described the use of MTA material in the treatment of teeth with horizontal root fractures [13, 14].

This case report describes the use of MTA in the multidisciplinary management of a patient with a horizontally fractured central incisor and luxation in a different central incisor.

## 2. Case Report

**Figure 1:**
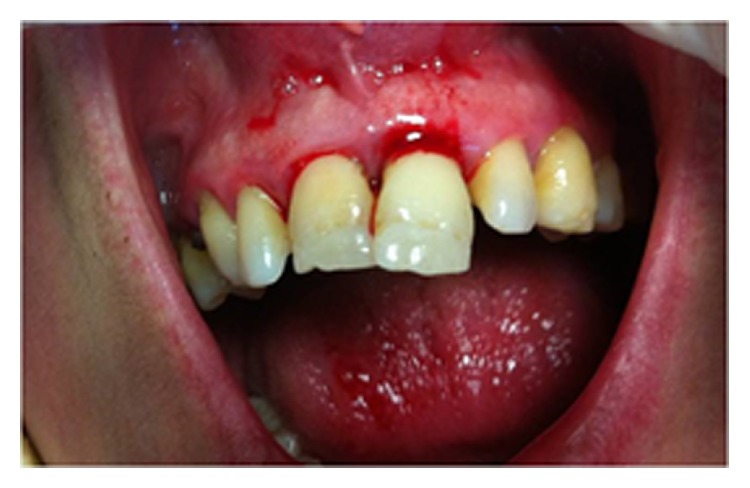
The intraoral view of the patient before treatment.

A 42-year-old female patient presented within 1 h of receiving direct trauma to her maxillary area. The medical history of the patient revealed no systemic disease. Clinical examination revealed that the right and left maxillary central incisors presented mobility (grade 2) and sensitivity to percussion and palpation but no sensitivity to thermal stimulations. Occlusal displacement with extrusion in the left maxillary central incisor and luxation in the right maxillary central incisor was observed. The crown of the left maxillary central incisor was slightly dislocated in the buccal direction. The remaining anterior incisors were clinically normal, and no signs of alveolar bone fracture were detected; however, gingival bleeding was present (Figure 1).

**Figure 2:**
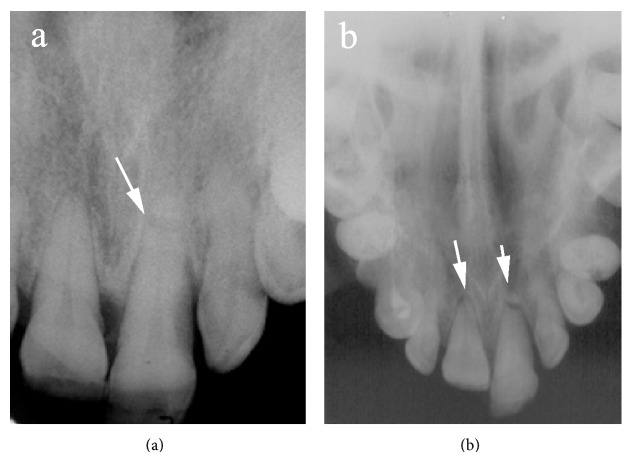
The radiographic view of the patient before treatment. (a) Arrow marks the horizontal root fracture. (b) Arrow marks the enlarged periodontal space of right and left maxillary teeth.

Two periapical radiographs with different vertical angles of the maxillary anterior teeth and an additional occlusal radiograph were taken. Radiographic examination revealed horizontal root fracture at the apical third of the left maxillary central incisor. Root fracture in the right maxillary incisor was not observed. The periodontal spaces of both maxillary central incisors were enlarged (Figures 2(a) and 2(b)).

**Figure 3:**
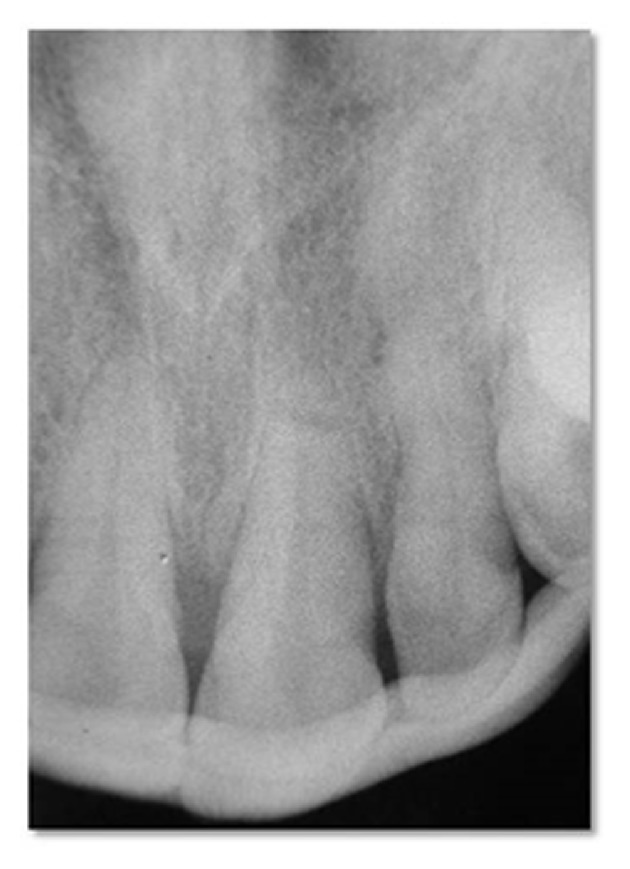
Repositioned coronal fragment and applied rigid splint.

The treatment plan comprised reduction, repositioning, and rigid splinting of the coronal fragment of the left maxillary central incisor. Following administration of local anesthetics, the coronal fragment was repositioned with digital pressure and the success of repositioning was confirmed radiographically before the rigid splint was applied with composite resin material (Filtek Supreme XT, 3M Espe, USA) (Figure 3). The patient was instructed to maintain good oral hygiene, follow a soft diet, and avoid masticating in that area. Right and left maxillary teeth were examined at 2-week intervals for 2 months.

At the end of the second month, the patient presented with a complaint of severe pain right and left maxillary teeth. Clinical examination revealed that these teeth showed pain to percussion and palpation. Sensitivity to thermal stimulations was not observed. It was decided to continue with endodontic treatment.

After isolating the teeth with a rubber dam, the access cavities were prepared, the necrotic pulps were extirpated, and the working lengths were obtained. Apex locator (Morita, Kyoto, Japan) was used in determination of the working length and was confirmed by radiographs. Left and right maxillary central incisors working lengths were, respectively, 17 and 20 mm. The root canals were prepared with nickel titanium rotary system (ProTaper Universal, Dentsply Maillefer, Ballaigues, Switzerland) until the finisher F4 instrument was completed. 5.25% sodium hypochlorite (NaOCl) irrigation solution was used for the cleaning and shaping procedures. After instrumentation, the root canal was flushed with 5 mL of 5.25% NaOCl and 5 mL distilled water, respectively, and dried with paper points. Calcium hydroxide paste was applied in the root canal space and the cavity was temporarily filled with temporary restoration (Cavit-G, 3M Espe, USA). The patient was scheduled for follow-up after 2 weeks.

**Figure 4:**
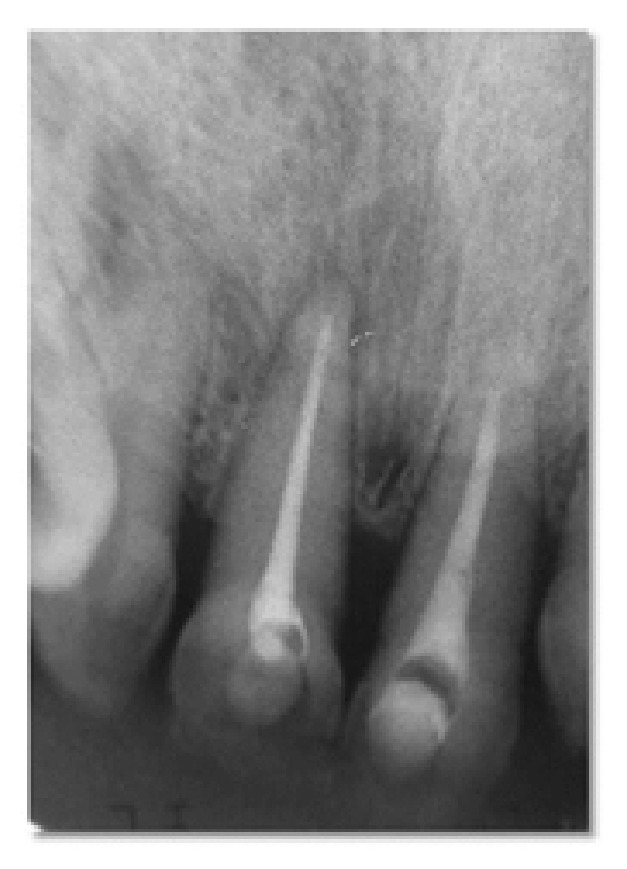
The radiographic view of the patient after root canal treatment.

At the next visit, clinical and radiographic examinations revealed no signs of pulpal infection in either of the maxillary incisors; therefore, the root canals were prepared for obturation. Composite resin splint was removed from teeth. Then the calcium hydroxide paste was removed from the root canals and MTA (ProRoot MTA, Dentsply Maillefer, Ballaigues, Switzerland) was prepared according to the manufacturer's instructions. An endodontic plugger adequate (American Eagle, Missoula, MT, USA) for the length of the root canal was used and the stopper was fixed 1 mm short of the working length. MTA was inserted into the canal with Messing gun and compacted further with the plugger. Then a moistened cotton pellet was placed on the MTA surface, and the endodontic access cavity was filled with temporary restoration (Cavit-G, 3M Espe, USA) (Figure 4). During the following visit, the cotton pellet was removed, and the remaining portion of the tooth was filled with composite resin. Next, the patient was referred to a restorative clinic.

**Figure 5:**
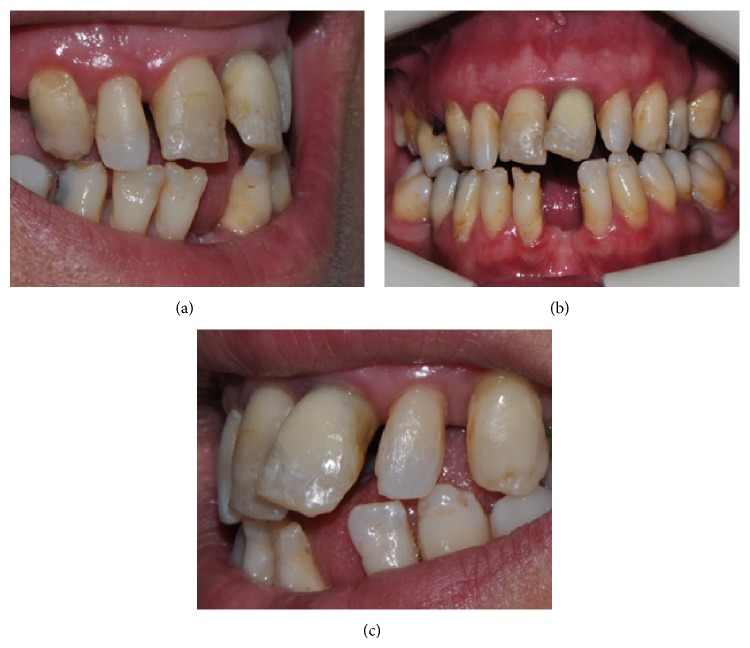
(a) Right lateral view before restorative treatment. (b) Frontal view before restorative treatment. (c) Left lateral view before restorative treatment.

The clinical oral examination for restorative treatment revealed the following aesthetic and appearance problems: malposition and displacement in left maxillary central incisor, unsuitable occlusion, unsuitable proportions and size of teeth, lingualisation of right maxillary canine and left maxillary first premolar teeth, absence of left mandibular central incisor, mal-texture and unsuitable appearance and colour of the teeth, and unequal and disproportionate gingival zenith level size of teeth (Figures 5(a), 5(b), and 5(c)).

Resin build-up and composite laminate veneers were planned for treatment of the mentioned aesthetic problems. Before any aesthetic treatments, all caries were removed and cavities, including endodontic coronal cavities, were restored with composite resin restorations (Filtek Supreme XT, 3M Espe, USA).

**Figure 6:**
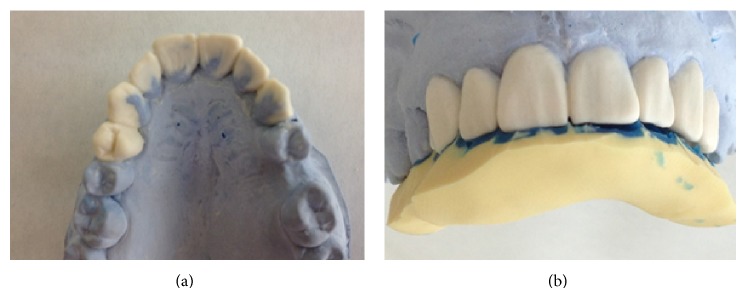
(a) Wax-up of the build-up of teeth on maxillary arch stone cast. (b) Silicone index to mark the palatal filling contour.

A wax model was fabricated, establishing an ideal incisal edge position and form. This ideal wax model defined the final three-dimensional tooth positions. A silicon index was made using the study cast to determine the index during composite treatment (Figures 6(a) and 6(b)).

Left maxillary central incisor was treated using composite build-up without an endodontic post. The other teeth were treated with direct composite veneer restorations performed using a polychromatic composite layering technique. Because A2 was the final restoration shade selected, the A3D shade (Filtek Supreme XT, 3M Espe, USA) was used to give the restoration a natural depth of shade (chroma) in the core of the filling. A2B and A1E shades (Filtek Supreme XT, 3M Espe, USA), which have medium transparency and chroma, were layered over this dentine shade to produce natural-looking shades. The C shade (Filtek Supreme XT, 3M Espe, USA), which has the greatest transparency and the least chroma, was used in combination with enamel and dentine shades to imitate the incisal edges. The buccal arch was labialised to achieve a better appearance of the buccal arch and reduce the carvings in the surface of the teeth. Pink coloured composite resin (Amaris Gingiva, Voco, Germany) was used to maintain the gingival embrasures and the gingival architecture of left maxillary lateral and canine.

**Figure 7:**
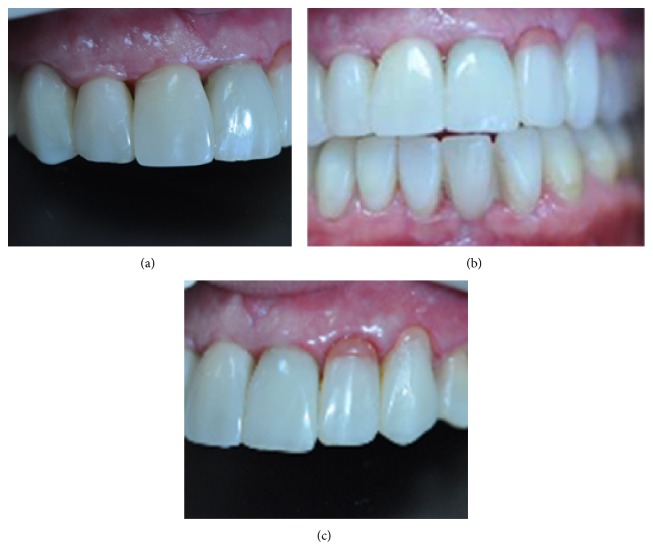
(a) Right lateral view after restorative treatment. (b) Frontal view after restorative treatment. (c) Left lateral view after restorative treatment. Pink color composite used at the edges of zenith level of 22 and 23.

A composite Maryland bridge cemented with dual cure composite resin cement (Panavia F2.0, Kuraray Medical Inc.) was used to permanently replace left mandibular central incisor and temporarily replace right maxillary first premolar. To improve the surface texture, the restorations were finished and polished with a mixture of coarse diamonds (837LH014, Meisinger, Germany), composite finishing carbides (HM245012, HM244010, Meisinger, Germany), and various grits of polishing discs (Sof-Lex, 3M Espe, USA) (Figures 7(a), 7(b), and 7(c)).

**Figure 8:**
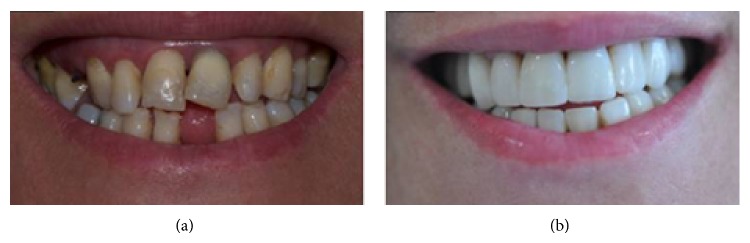
((a), (b)) Stretched smile before and after restorative treatment.

**Figure 9:**
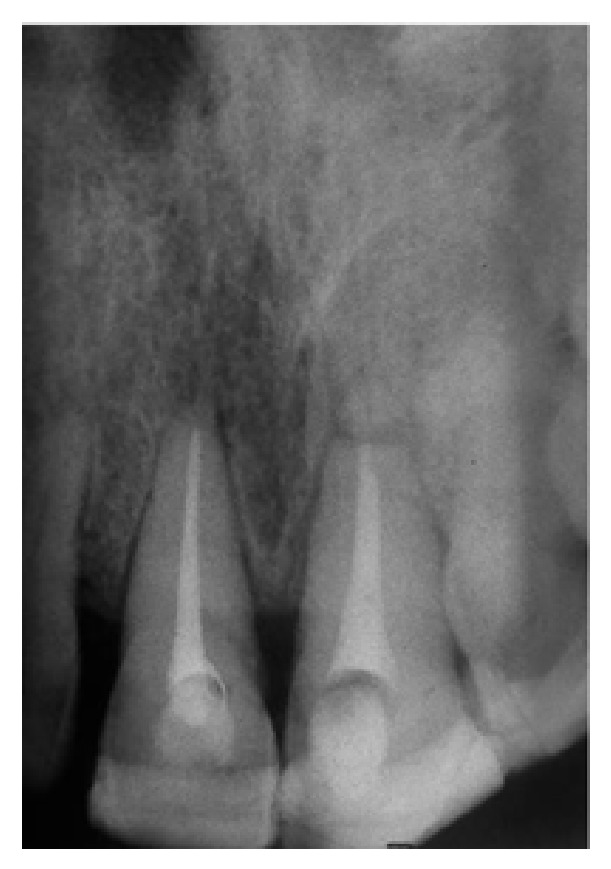
After 15-month radiographic control examination.

Endodontic and aesthetic restorative treatments were completed, and the patient's oral hygiene motivation was improved significantly (Figures 8(a) and 8(b)). The clinical condition of the restorations and the patient's oral hygiene were in good condition at the 15-month follow-up examination (Figure 9).

## 3. Discussion

Dental pulp necrosis occurs in 20–44% of root fracture cases; however, necrosis in luxated teeth without fracture is more common and occurs in at least 43.5% of cases. In the absence of clinical and/or pathological signs, emergency endodontic treatment is not recommended for root fracture and luxated teeth. In such cases, the teeth should first be followed up clinically and radiographically [6, 14–16]. Endodontic treatment should be initiated in teeth that do not respond to a vitality test (electrometric or thermal pulp test) in 3 months or that show radiolucency next to the fracture line. When pulp necrosis develops, the apical part of the fractured tooth usually remains vital; hence, root canal treatment is performed only in the coronal fragment [14]. In our case, in the beginning of the treatment planning, pulp sensitivity tests were planned to be performed every 3 months as applied in Westphalen et al. [2]. Öztan and Sonat [17] suggested that “immediate endodontic intervention should be avoided, mentioning that clinical and radiographic follow-up could be the treatment chosen, provided there are no clinical and radiographic signs of pathological alterations and there is pulp vitality.” In that respect, we avoided immediate endodontic treatment and conducted regular clinical examinations. We have conducted clinical examinations on a biweekly basis and clinical examination did not reveal any discoloration or any other clinical symptoms. However, at the end of the second month, the patient presented the clinic with a complaint of severe pain right and left maxillary teeth and considering patient's condition root treatment was performed at the end of the second month.

Several studies have reported that rigid splinting must be maintained for 2-3 months [3, 16]; however, the effect of the splinting period on prognosis has not yet been determined [18]. In our case, right and left maxillary teeth had severe mobility and dislocation, and the 2-month duration of the fixed appliance was considered safe and viable for healing.

Elimination of microorganisms from the root canal system determines the full success of endodontic therapy, particularly in cases of pulp necrosis and periradicular lesions [19, 20]. To increase the effectiveness of endodontic therapy, various intracanal dressings have been used as adjuncts; calcium hydroxide, one of the most common dressings [20, 21], was used in this case.

MTA is a biocompatible material that has shown great potential as an endodontic material [13, 14, 22–24]. First recommended as a material to repair root perforations, it has since been used widely as a root-end filling material. Additionally, it has been used for vital pulp therapy such as direct pulp capping and pulpotomy of immature teeth with vital pulp (apexogenesis). Furthermore, because of its sealing ability, it is used as an apical barrier in the treatment of teeth with opened apices and necrotic pulps (apexification) [22–25]. MTA adheres and conforms very precisely to the dentinal walls, thus providing the hydraulic seal [13].

Andreasen et al. [26] have shown that root canal filing with MTA placement of calcium hydroxide for disinfecting the dentinal tubules and root canal offered improved long-term fracture resistance. Torabinejad and Chivian [23] recommended the use of MTA in teeth with necrotic pulp and open apices and reported that MTA material was surrounded with new cementum formation. Yildirim and Gençoğlu [27] showed the management of two maxillary central incisors with horizontal root fractures using MTA in their cases.

The use of MTA in the treatment of horizontal root fractures is not a routine application; however, considering the advantages explained above, we decided to use MTA in our treatment and MTA positively affected the healing of fractured teeth.

In the restoration of traumatised anterior teeth, both aesthetic and mechanical considerations should be taken into account. For this case, there were two treatment alternatives: composite resin restorations and prosthetic treatment. Composite resin restorations were chosen for this patient, considering the advantages of lower treatment time and minimal tissue removal. After treatment plan was discussed with the patient, composite resin restorations were chosen, considering the advantages of lower treatment time and minimal tissue removal. The clinical technique is noninvasive and reversible so that all other restorative options can be evaluated at a later date. It offers a simple and cost-effective treatment option [28].

## 4. Conclusion

Within the limitations of this case, MTA showed a good long-term outcome when used in root-fractured and luxated teeth. In addition, composite resin restoration provided satisfactory aesthetic results even after 15 months. The patient was scheduled for follow-up visits at 1-year intervals in 5 years.
